# Tobacco, Nicotine and Health

**DOI:** 10.3390/medicina57080740

**Published:** 2021-07-22

**Authors:** Manfred Neuberger

**Affiliations:** 1 Commission on Climate and Air Quality, Austrian Academy of Science, 1010 Vienna, Austria; manfred.neuberger@meduniwien.ac.at; 2 Center for Public Health, Medical University of Vienna, 1090 Vienna, Austria

**Keywords:** tobacco, nicotine, smoking, vaping, addiction, prevention, cessation, health, Europe

## Abstract

Recent studies have explored improvements in smoking prevention and cessation to reduce smoking prevalence, however, in Europe, only Finland has already set a date to become nicotine free. Studies reporting on central, eastern and southern Europe have mostly focused on combustible cigarettes up to now. In young people, correlations were found between traditional smoking, the “vaping” of e-cigarettes, experimentation with alcohol intoxication, and the use of illicit drugs. Prevention and cessation should include strategies against active and passive exposures to new nicotine products. This is a prerequisite for a successful public health policy and a future end-game against the business interests of the tobacco industry and its allies.

This Special Issue of MDPI *Medicina* (section “Epidemiology & Public Health”) reports new studies from Europe on the prevention and cessation of smoking. Some Western countries have already prepared for “end-game strategies” and Finland has set a date to become nicotine-free; however, in some central, eastern and southern European countries, smoking prevalence remains high and tobacco control is insufficient, focused on combustible cigarettes, and the international tobacco industry remains very successful with all their products [1,2]. Budin et al. [3] studied the smoking behavior of abandoned children and teenagers and found that they started to smoke earlier than those in normal familiar environments; however, a professional maternal assistance system helped against the passive and active smoking of adolescents. Sanchez et al. [4] found a lack of physical activity in persons with asthma aged 15–69 years, but in healthy girls of 14–16 years of age, Maric et al. [5] detected interaction between the physical activity level and non-smoking, which could be used in prevention. In boys of that age, physical activity alone was insufficient to prevent smoking. Lotrean et al. [6] found significant correlations in university students between e-cigarette use, smoking, experimentation with alcohol intoxication, and the use of illicit drugs. This underlines the necessity for prevention that also includes e-cigarette use. Multiple positive effects could be expected from addiction prevention and early nicotine cessation. Haluza et al. [7] asked general practitioners on smartphone use by lung patients, concluded that the use of apps should be part of health care and recommended apps for cessation to smokers of all ages.

The WHO fights health risks from tobacco [8] and new nicotine products [9], against business interests of a mighty industry. This business is based on nicotine addiction and uses the seduction of adolescents, the manipulation of public opinion by advertisements, the sponsoring and promotion of deadly cigarettes and the undermining of public health policy by misleading information on nicotine products [10,11,12]. Recently, the tobacco industry has diversified to make profit from tobacco and its so-called “substitutes”, sold as cessation aids. In fact, such “substitutes” serve as gateway drugs into nicotine addiction for young people and other new customers, leading to dual and multiple uses of nicotine products, which increase health risks and make it more difficult for the customer to stop using nicotine products [13,14,15,16]. This strategy is masked as “harm reduction”, however, all innovations and promotions of filter cigarettes, “light cigarettes”, water pipes, e-cigarettes and heated tobacco to date have resulted as harmful for customers and are only beneficial for the business of the tobacco industry, its retailers and allies [9,10,11,12,13]. Most recently, the largest tobacco company attempted a takeover of inhalation drugs by British Ventura^®^ and oral drugs and chewing gum by Danish Fertin^®^ to enter the pharma market [17]. In contrast to nutrition products on the free market, the control of pharmaceuticals for the treatment of disease has become much better to date. However, this is endangered by a mix-up of addictive substances with luxury foodstuffs, stimulants, enjoyment spices, etc., and even with pharmaceuticals from an industry with a history of corruption and fraud. Selling NRT without prescription was possibly already a mistake [11]. However, most dangerous would be an “elephant marriage” of Big Tobacco with Big Pharma, an uncontrolled diversification of nicotine products and other addictive substances, advertised as lifestyle products for well-being, including the promotion of gateway drugs for children on the free market.

We need more regulation for tobacco and nicotine products, particularly nicotine products destined for inhalation. The prevention of nicotine addiction has been more successful in the long term than smoking cessation, however, for a quick reduction in diseases and mortality from tobacco and related products, efforts to improve nicotine cessation must also be enforced, particularly for the cessation of combustible tobacco products. Both active and passive exposures to traditional and new nicotine products and their health effects [9,10,11,12,18,19,20] need to be reduced. This is a prerequisite for a successful public health policy and a future end-game against the business interests of the tobacco industry and its allies.

## Data Availability

See references.
